# A novel method of evaluating the non-invasive tear film break-up time and progression of corneal opacification in dogs using imaging video

**DOI:** 10.3389/fvets.2024.1298467

**Published:** 2024-04-08

**Authors:** Suk Jun Lee, Myeong Gyun Han, Su-Jung Yang, Yun-Soo Choi, Joon Young Kim

**Affiliations:** ^1^Division of Business Administration, College of Business, Kwangwoon University, Seoul, Republic of Korea; ^2^Department of Veterinary Ophthalmology, College of Veterinary Medicine, Konkuk University, Seoul, Republic of Korea; ^3^KU Center for Animal Blood Medical Science, Konkuk University, Seoul, Republic of Korea

**Keywords:** non-invasive break-up time, tear film break-up time, ocular surface disease, dry eye disease, corneal opacification

## Abstract

This study aimed to determine the correlation of the parameters that indicate the status of the ocular surface with the prognosis of corneal opacification. Fifty dogs (96 eyes) were examined using a grid-line illuminator (non-invasive tear film break-up time (NIBUT)). Thirty dogs (54 eyes) were included in the final analysis based on the criteria. The NIBUT and tear film break-up time (TFBUT) results of the eyes included in the study were divided into three groups: Group 1 (< 5 s), Group 2 (5 to <10 s), and Group 3 (≥ 10 s). The Schirmer’s tear Test 1 (STT-1) results of the included patients were also divided into three groups: Group 1 (< 5 mm/min), Group 2 (5 to <10 mm/min), and Group 3 (≥ 10 mm/min). The corneal opacity grades are divided into four scores, ranging from 0 to 3. The corneal opacity grade score (COS) of 0 indicates a completely clear cornea or only a trace of opacity. COS of 1, 2, 3 indicate the presence of a prominent corneal opacity that does not interfere with the visualization of the fine iris details, the opacity obscures the visibility of the iris and lens details and severe obstruction of the intraocular structure visibility, respectively. The mean difference in COS during the follow-ups for each group of NIBUT were 0.61 ± 0.92 (*n* = 28), 0.10 ± 0.32 (*n* = 10), 0.19 ± 0.40 (*n* = 16). The NIBUT groups were significantly correlated with COS (*p*-value = 0.073) at a 10% level of significance. Post-hoc test at a 10% level of significance revealed significant correlations between Groups 1 and 2 (*p*-value = 0.041) and between Groups 1 and 3 (*p*-value 0.104). Although the TFBUT and STT-1 groups did not show any significant correlation with COS. Eyes with NIBUT of <5 s were found to have a significantly higher chance of increased COS compared with eyes with NIBUT of >5 s in the grid-line illumination plate NIBUT test. Among NIBUT, STT-1, and TFBUT, NIBUT was the only test that showed significant associations with the changes in COS.

## 1 Introduction

The ocular surface and ocular diseases contribute to the status of the ocular surface. Inflammation, irritation, and corneal surface opacification affect the quality of life. The ocular surface includes the tear film, conjunctiva, cornea, eyelids, and lacrimal glands. Therefore, although the term ocular surface disease (OSD) is often used synonymously with dry eye disease (DED) in human medicine, the clinical aspects of DED belong to a group of OSD that also includes other diseases, such as blepharitis, meibomian gland dysfunction, allergic eye diseases, and other conditions, affecting the ocular surfaces. Diseases affecting these structures cause OSD, and patients often present with clinical signs of ocular irritation, redness, and reduction of visual acuity (1, 2).

The term DED is synonymous with the term “keratoconjunctivitis sicca” (KCS) in veterinary medicine. Currently, the sole standard criterion used for diagnosing KCS is the Schirmer’s tear Test 1(STT-1). However, as it does not encompass the entire concept of DED, studies have aimed to develop further diagnostic methods (3–6). Assessment of ocular surface health is vital for the early detection of early DED. The non-invasive tear film break-up time (NIBUT) test, classical fluorescein staining tear film break-up time (TFBUT) test, and the magnified view under slit-lamp biomicroscopy are commonly used methods to evaluate the corneal surface and tear film. Additional examinations, such as STT-1, intraocular pressure tonometry, optical coherence tomography (OCT), *in vivo* confocal microscopy, meibography, and interferometry, have also been used to further assess the tear film status (7, 8).

Many diagnostic techniques, such as NIBUT, TFBUT, meibometry, tear osmolarity, and interferometry, focus on the stability of the tear film. Tear film stability refers to the rate of evaporation of the tear film. The tear film constitutes many components, namely the superficial lipid layer, the overlapping and interchanging aqueous layer, and the mucin layer. These components work together to create a stable tear film. Diseases, environmental factors, and other factors, such as age and history of ocular surgery, may cause excessive break-up of the tear film (1).

Several studies have been conducted on the results of other diagnostic techniques used for the detection of DED in veterinary medicine; however, a standard has not yet been established (4–6, 9–12). One of these diagnostic techniques, NIBUT, was first introduced in human medicine by Mengher et al. (13). It is a non-invasive, dye-free method that has been widely used in clinical practice. The NIBUT systems commonly used in veterinary medicine are implemented directly from human medicine; however, the lack of standard criteria has increased the need for novel approaches using imaging-based diagnostic testing. No previous study has focused on the correlation between the progression of the disease and the diagnostic test results, which may reflect the prognosis of the disease at a certain stage based on the test result. A modified version of the original NIBUT system developed by Mengher et al. (13), which sheds a grid-shaped illumination on the corneal surface, has been used as the diagnostic method to evaluate the status of the ocular surface in this study. Previous studies on the use of grid-line illumination NIBUT system utilized an add-on illuminator plate over the commonly distributed ophthalmic slit-lamp biomicroscopes (14). The square grid lines of light reflected on the corneal surface represent the precorneal tear film (PTF), and the disturbance or disruption of these lines represents the break-up of the tear film (14).

This study aimed to evaluate the status of the ocular surface and compare the changes in corneal opacity over the course of treatment to determine the correlation between the status of the ocular surface and the prognosis of corneal opacification.

## 2 Materials and methods

Among the patients who visited the Veterinary Medical Teaching Hospital (VMTH) at Konkuk University between August 5th, 2020, and October 1st, 2021, 50 dogs (96 eyes) underwent examinations using the grid-line illuminator (NIBUT). The VMTH, Konkuk University requests the owners of the animals enrolled in a study to provide a patient consent form as a routine procedure. This form notifies the owners that patient information obtained during treatment may be used for research purposes. The study was approved by the Institutional Animal Care and Use Committee of Konkuk University (protocol no.: KU20123). All pet owners provided written informed consent.

**Figure 1:**
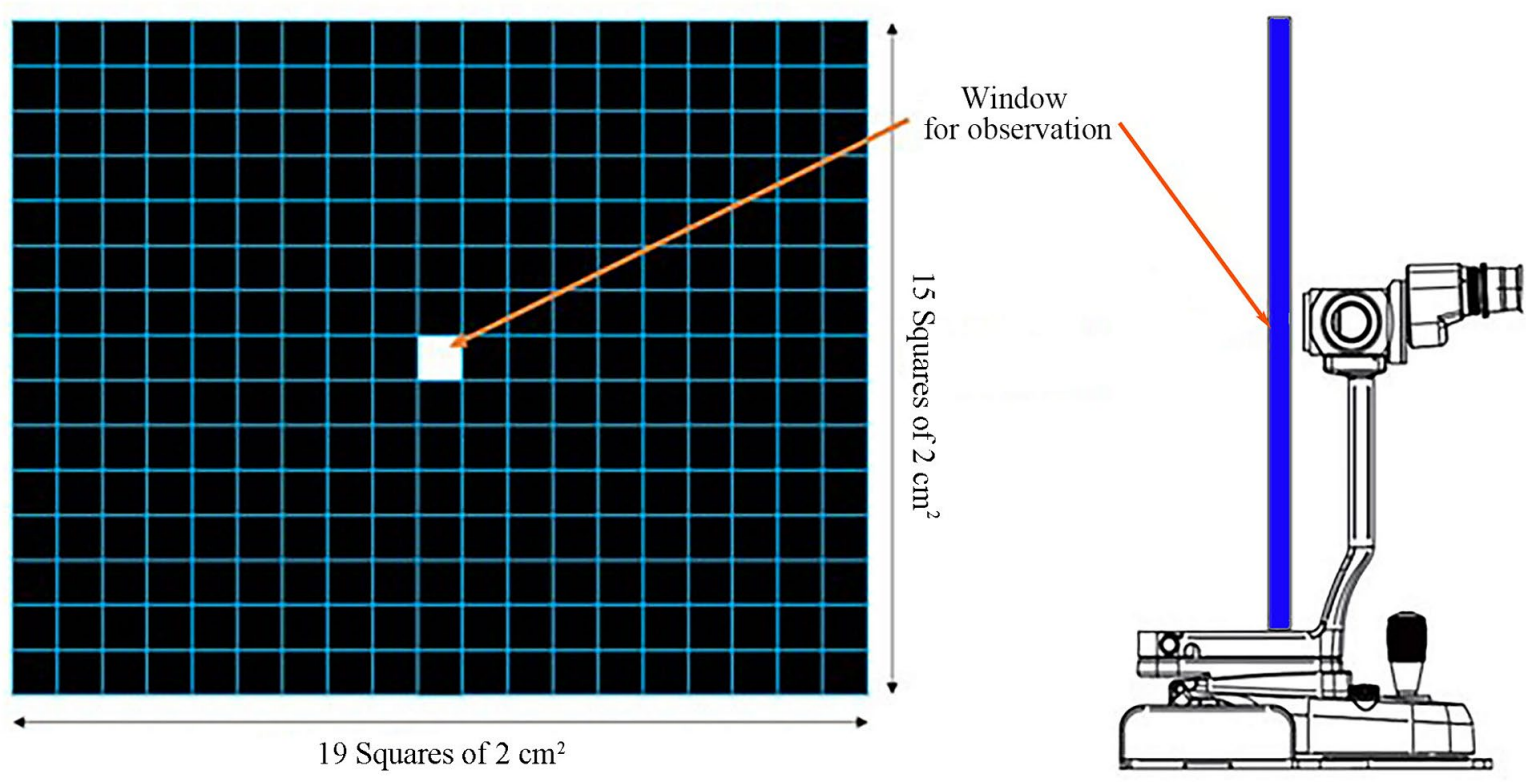
Grid-line illuminator for evaluating the non-invasive tear film break-up time (NIBUT). The grid plate was 42 cm × 33 cm in dimension corresponding to a total of 19 squares × 15 squares of 2 cm^2^ grid square lines. A window for observation via the slit-lamp is located at the center (14).

**Figure 2:**
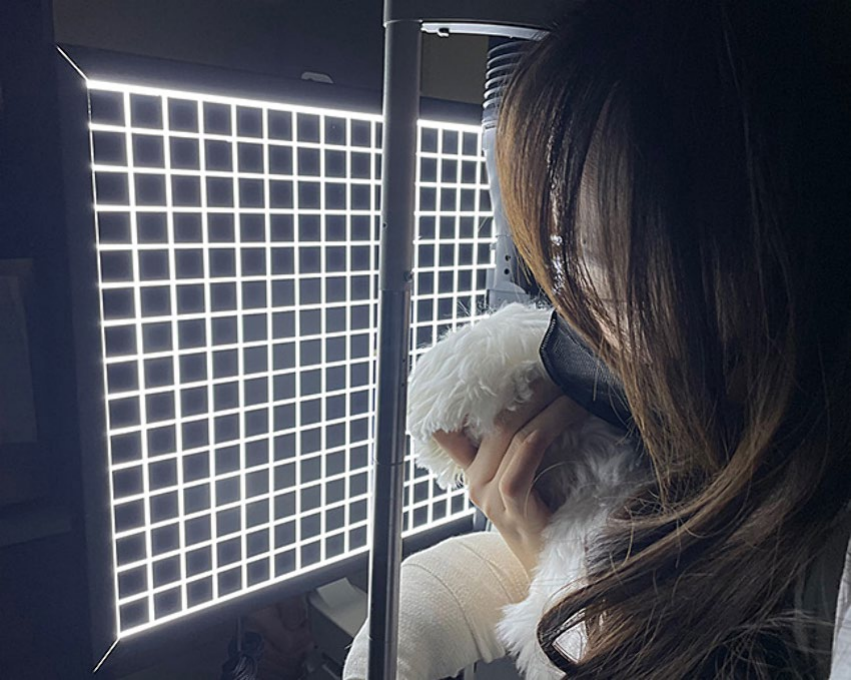
Photograph of the Grid-line illuminator utilized for the non-invasive tear film break-up time (NIBUT) examination in action.

The following method was used to evaluate NIBUT with the grid-line illuminator. Slit-lamp biomicroscope (GS LED Slit Lamp MW50D, Shigiya machinery works LTD., Japan) with an add-on custom-made grid-plate illuminator (a 42 cm × 33 cm plate with LED lights corresponding to a total of 19 squares × 15 squares of 2 cm^2^ in size grid) was used to obtain a 20-s video of images from all patients (Figures 1, 2) (14). All videos were recorded prior to conducting any of the other examinations. Recording of the video was commenced once the assistant opened the eyes of the patients with gentle force. The patients were minimally restrained to prevent blinking during the examination. NIBUT video recording continued until a clear image that could be judged objectively was obtained, and the examination concluded upon acquiring a 20-s video suitable for grid line evaluation. Images that failed to cover the entire cornea were excluded. All tests were performed in one room with the temperature set between 20°C to 25°C and humidity between 30 and 40%. All sources of ventilation were turned off before the examination to reduce interference from factors that may affect the test results. All images were analyzed by a single observer (M.G.H) to minimize the risk of subjectivity bias that may occur if the analyses are performed by multiple examiners. A count-up timer was started from the start of the video and stopped as soon as the observer detected any break-up or distortion of the straight grid lines.

Fluorescein-staining TFBUT test was performed by dissolving a sterile strip of 1 mg of fluorescein in 0.2 mL of sterile 0.9% sodium chloride solution, which was then instilled on the ocular surface. The patients were permitted to blink a few times, and the count-up timer was started as the eyelids were opened with gentle force and examined under cobalt-blue light. The time at which the examiner detected the break-up of the fluorescein-stained tear film was marked for each patient. Tear film Break-up is defined as a dry spot in the dye-stained tear film wherein the green light is disrupted, revealing dark spots without tear film (15–17). This process was repeated a total of three times, and the average value was calculated.

**Figure 3:**
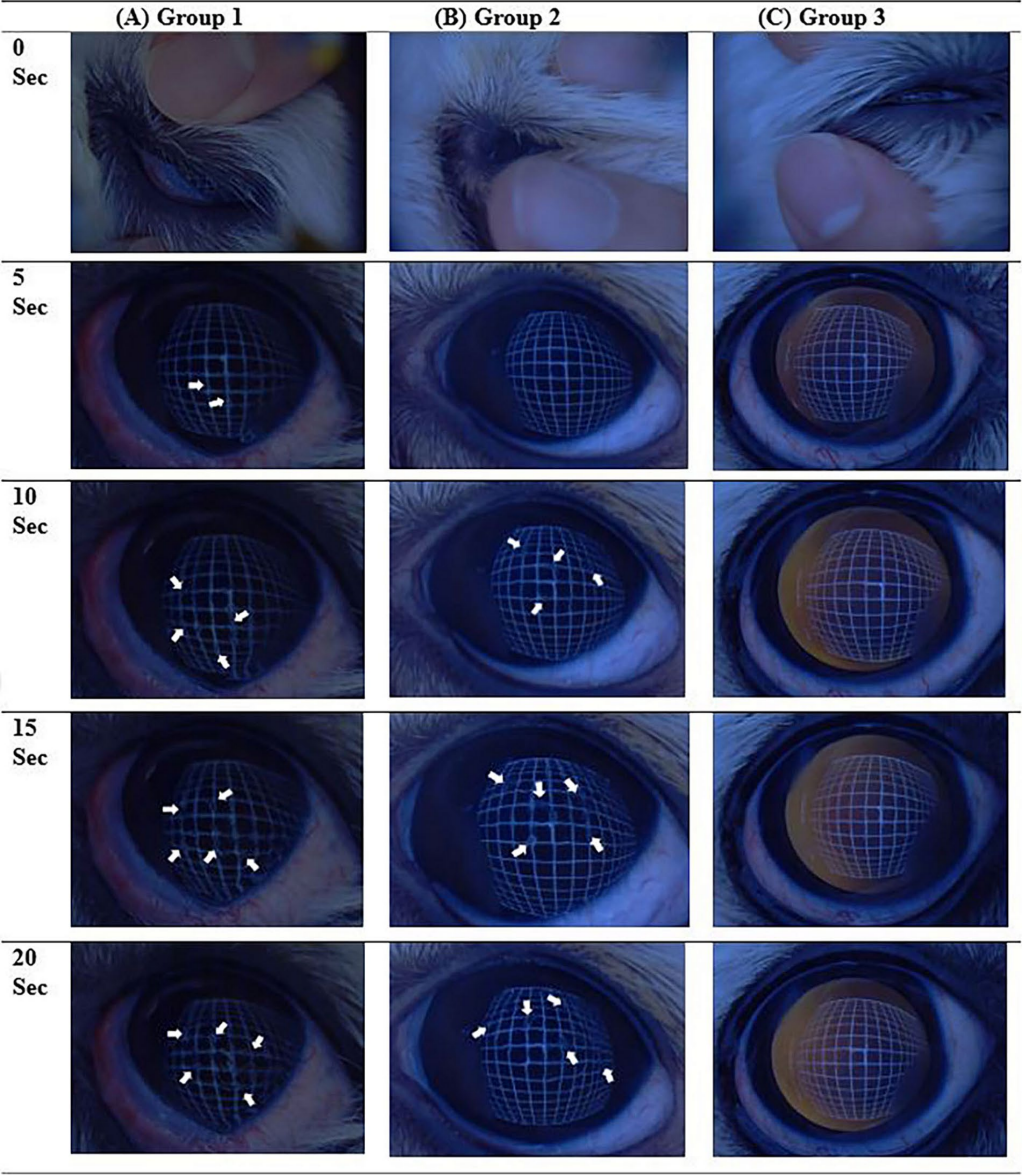
Examples of the non-invasive tear film break-up time (NIBUT) video images examined, showing a series of images every 5 s for each group. **(A)** Case number 18 of NIBUT group 1, where all images beyond 5 s contain interrupted grid lines (arrows). **(B)** Case number 29 of NIBUT group 2, with images beyond 10 s showing interrupted grid lines (arrowheads). **(C)** NIBUT group 3, demonstrating images with no interruption of grid lines up to 20 s.

The numerical value of the results of the NIBUT tests were compared with those of human medical reports. As glaucomatous eyes may affect the results of corneal opacity, patients with uncontrolled intraocular pressure (IOP) were excluded from the study. Patients who met the following criteria were included in this study: IOP did not exceed 20 mmHg after the administration of the first antiglaucoma drugs and absence of corneal opacification after returning to the baseline IOP. Similarly, patients who were suspected to have corneal endothelial diseases, which are likely to progress to corneal opacification, were also excluded. According to the reports in human medicine, the cut-off value for normality of the TFBUT and NIBUT results is >10 s, with values <5 s indicating severe DED (17, 18). The results of NIBUT and TFBUT of the patients included in this study were divided into three groups: the severe group (designated as Group 1, with a tear film break-up time of <5 s), the mild group (Group 2, with a tear film break-up time of 5–10 s), and the normal group (Group 3, with a tear film break-up time of >10 s). The STT-1 results of the patients included in this study were also divided into three groups: the severe group (Group 1, value less <5 mm/min), the moderate group (Group 2, value within 5–10 mm/min), and the normal group (Group 3, value >10 mm/min) (Figure 3).

All patients underwent complete ophthalmic examinations, such as STT-1, IOP, and slit-lamp biomicroscopy. The medical history and follow-up data of the patients were gathered to assess the changes in corneal opacification according to the corneal opacity grading system used in human medicine. The corneal opacity grading was determined using slit-lamp biomicroscopy. The grades are divided into four scores, ranging from 0 to 3, based on the system reported by Fantes et al. (19). The corneal opacity grade score (COS) of 0 indicates a completely clear cornea or the presence of a trace of opacity. A COS of 1 indicates the presence of a prominent corneal opacity that does not interfere with the visibility of the fine iris details. A COS of 2 indicates the presence of an opacity that obscures the vis of the iris and lens details. A COS of 3 indicates severe obstruction of the visibility of the intraocular structure. Data regarding the initial (at the time of NIBUT examination) corneal opacity grade scores, NIBUT results, and TFBUT results of the patients were collected. The follow-up data contained COS. The initial COS refers to the score measured at the time of the NIBUT examination, whereas the end-point COS refers to the highest COS measured during the follow-up visits. The end-point COS value was adopted from the last examination at least 150 days after the first test. Patients with iatrogenic corneal damage, such as those who underwent intraocular surgery, were excluded from the scoring process for 1 month of time as a time-out due to the healing of the corneal wound that may reduce the corneal opacity.

The data were analyzed using SPSS software (Statistical Package for the Social Sciences version 28 for a window; IBM). One-way analysis of variances (ANOVA) was used to evaluate the changes in COS during the follow-up visits, the difference in COS at the end-point, and the differences between the NIBUT, TFBUT, and STT-1 results. ANOVA is a statistical method used to compare the means of three or more independent groups to determine if there exists a statistically significant difference among them. It is an extension of the t-test, which is used for comparing means between two groups. The “one-way” aspect refers to the analysis being conducted on a single independent variable categorizing the groups. Subsequently, depending on the assumption of homoscedasticity, the Games–Howell test and Bonferroni post-hoc test were performed to confirm these results. Post-hoc tests are necessary for drawing valid inferences about specific group differences subsequent to an initial ANOVA indicating overall differences among groups. They provide a methodological approach to pinpoint where the differences lie, thereby ensuring the reliability and validity of statistical conclusions. In addition, a comparison of mean differences between the results of NIBUT, TFBUT, and STT-1 was conducted using ANOVA.

## 3 Results

The study cohort comprised 28 castrated males (CM), three intact males (IM), 16 spayed females (SF), and three intact females (IF). Forty-eight right eyes (OD) and 48 left eyes (OS) were evaluated in this study. The study population comprised Poodles (*n* = 14), Maltese (*n* = 12), Shih-Tzu (*n* = 8), and other breeds, such as Bichon Frise, Chihuahua, Cocker Spaniel, Coton de Tulear, Miniature Pincher, Spitz, Pug, and Yorkshire Terrier. The average age of the patients was 7.80 ± 3.65 years (average ± standard deviation).

**Figure 4:**
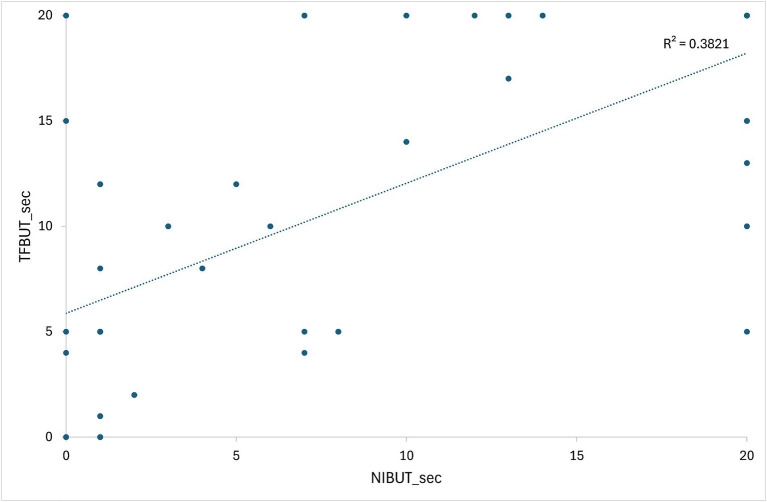
Illustration depicting the correlation between values obtained from NIBUT and TFBUT.

**Figure 5:**
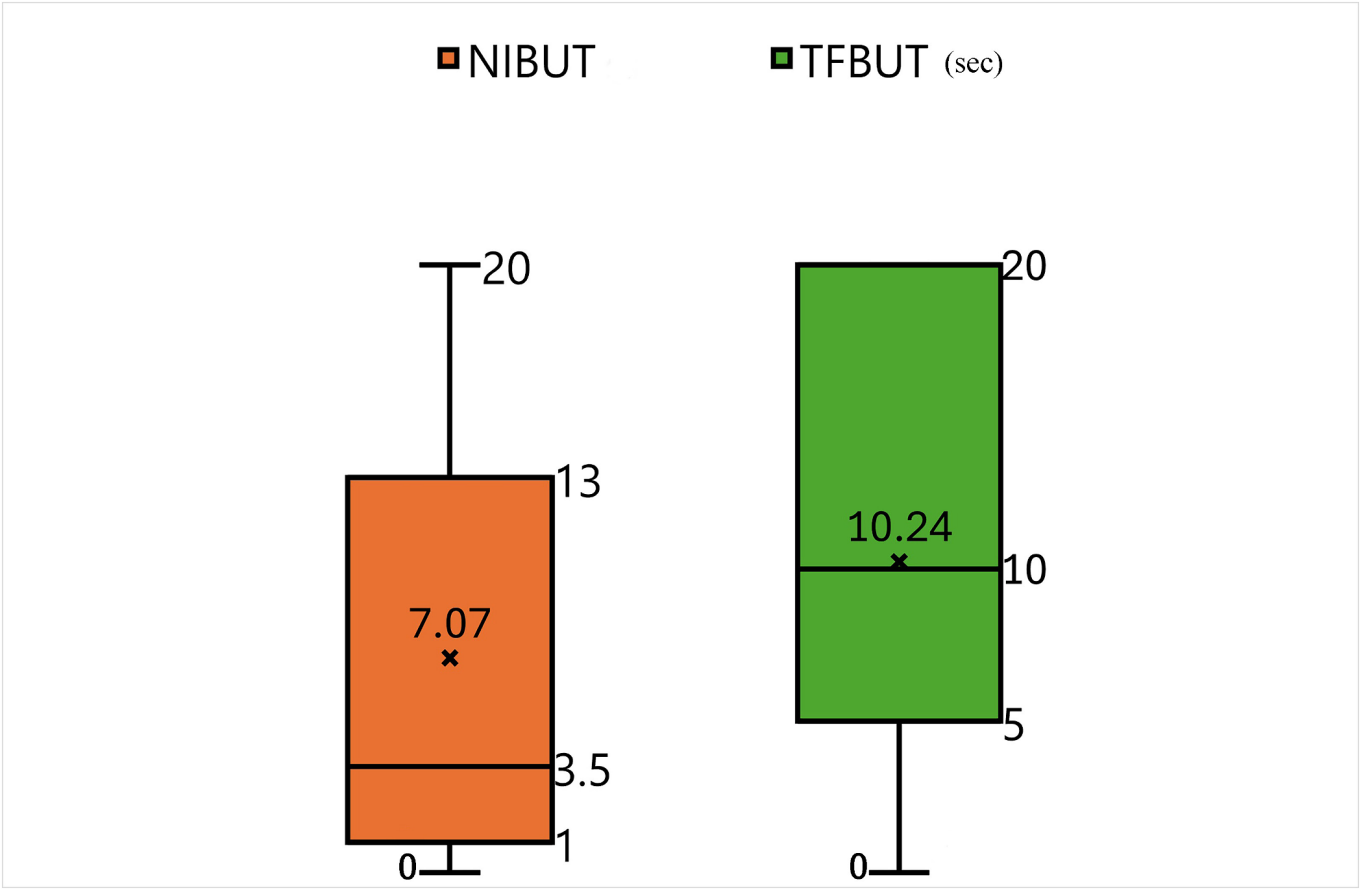
Descriptive statistics analyzed using values from NIBUT and TFBUT.

Supplementary Table S1 presents the initial data of the results of STT-1, NIBUT, and fluorescein TFBUT tests, along with the COS during the initial and end-point examinations. Cases not meeting the criteria (total 20 patents) were excluded from data analysis. For example, case 22 was excluded due to a history of acute severe corneal chemical injury resulting from the use of shampoo. Consequently, 30 patients (54 eyes) were evaluated after applying the inclusion criteria (Supplementary Table S1). All 30 patients (54 eyes) who re-visited for regular check-ups and follow-ups were evaluated during each follow-up visit to determine the COS. The average duration between two COS examinations (54 eyes) was 277.70 ± 109.38 days. Figure 4 illustrates the correlation between values measured by NIBUT and TFBUT, while Figure 5 provides a description of the statistical analysis results of NIBUT and TFBUT.

The slit-lamp microscopic images acquired at each follow-up visit were analyzed to measure the COS. Patients with corneal wounds did not undergo imaging for a minimum period of 2 weeks to exclude images showing acute corneal opacification that may resolve as the corneal wound heals. The mean difference in COS during the follow-up visits in each group, i.e., Groups 1, 2, and 3, of NIBUT were 0.61 ± 0.92 (*n* = 28), 0.10 ± 0.32 (*n* = 10), and 0.19 ± 0.40 (n = 16), respectively. The mean difference in COS for the entire study population was 0.39 ± 0.74 (*n* = 54). The NIBUT groups showed a significant correlation with COS (ANOVA, *p*-value = 0.073) at a 10% level of significance. The Games–Howell post-hoc test at a 10% level of significance revealed significant correlations between Groups 1 and 2 (*p*-value = 0.041) and between Groups 1 and 3 (*p*-value 0.104). No significant correlation was observed between Groups 2 and 3 (*p*-value 0.813) (Table 1).

**Table 1:** Results of (a) ANOVA and (b) the Games–Howell post-hoc test for the changes in COS and NIBUT.

(a) Results of ANOVA					
	Sum of squares	Degrees of freedom	Mean square	*F*	*p*-value
Between NIBUT Groups	2.817	2	1.409	2.761	0.073
Within NIBUT Groups	26.016	51	0.510		
Total	28.833	53			

(b) Results of the Games–Howell post-hoc test					
Dependent variable	(I) NIBUT groups	(J) NIBUT groups	Mean difference (I-J)	Standard error	*p*-value
Changes of COS	1	2	0.507^*^	0.200	0.041
		3	0.420	0.200	0.104
	2	1	−0.507^*^	0.200	0.041
		3	−0.088	0.142	0.813
	3	1	−0.420	0.200	0.104
		2	0.088	0.142	0.813

^*^The mean difference is significant at the 0.1 level. NIBUT, non-invasive tear film break-up time, COS, corneal opacity grade scores.

The mean difference in COS during the follow-up visits in each group, i.e., Groups 1, 2, and 3, of TFBUT were 0.58 ± 0.90 (*n* = 12), 0.22 ± 0.43 (*n* = 18), 0.42 ± 0.83 (*n* = 24), respectively. The mean difference in COS for the entire study population was 0.39 ± 0.74 (*n* = 54). The TFBUT groups did not show any significant correlation with COS (ANOVA, *p*-value = 0.417). The Bonferroni post-hoc test at a 10% level of significance revealed no significant correlation between Groups 1, 2, and 3 (Table 2).

**Table 2:** Results of (a) ANOVA and (b) the Bonferroni post-hoc test for the changes in COS and TFBUT.

(a) Results of ANOVA					
	Sum of squares	Degrees of freedom	Mean square	*F*	*p*-value
Between TFBUT Groups	0.972	2	0.486	0.890	0.417
Within TFBUT Groups	27.861	51	0.546		
Total	28.833	53			

(b) Results of the Bonferroni post-hoc test					
Dependent variable	(I) TFBUT groups	(J) TFBUT groups	Mean difference (I-J)	Standard error	*p*-value
Changes in COS	1	2	0.361	0.275	0.587
		3	0.167	0.261	1.000
	2	1	−0.361	0.275	0.587
		3	−0.194	0.230	1.000
	3	1	−0.167	0.261	1.000
		2	0.194	0.230	1.000

TFBUT, tear film break-up time; COS, corneal opacity grade scores.

The mean difference in COS during the follow-up visits for each group, i.e., Groups 1, 2, and 3, of STT-1 were 0.5 ± 0.71 (*n* = 2), 0.33 ± 0.58 (*n* = 3), 0.39 ± 0.76 (*n* = 49), respectively. The mean difference in COS for the entire study population was 0.39 ± 0.74 (*n* = 54). The STT-1 groups showed no significant correlation with COS (ANOVA, *p*-value = 0.970). The Bonferroni post-hoc test at a 10% level of significance revealed no significant correlation between Groups 1, 2, and 3 (Table 3). The mean difference between the results of NIBUT, TFBUT, and STT-1 was evaluated using ANOVA. The results of the tests were significantly correlated with the tests (*p*-value = 0.000). The Games–Howell post-hoc test at a 10% level of significance revealed significant correlations between NIBUT and TFBUT (*p*-value = 0.076), between NIBUT and STT-1 (p-value 0.000), and between TFBUT and STT-1 (*p*-value = 0.000) (Table 4).

**Table 3:** Results of (a) ANOVA and (b) the Bonferroni post-hoc test for the changes in COS and STT-1.

(a) Results of ANOVA					
	Sum of squares	Degrees of freedom	Mean square	*F*	*p*-value
Between STT-1 Groups	0.034	2	0.017	0.030	0.970
Within STT-1 Groups	28.799	51	0.565		
Total	28.833	53			

(b) Results of the Bonferroni post-hoc test					
Dependent variable	(I) STT-1 groups	(J) STT-1 groups	Mean difference (I-J)	Standard error	*p*-value
Changes in COS	1	2	0.167	0.686	1.000
		3	0.112	0.542	1.000
	2	1	−0.167	0.686	1.000
		3	−0.054	0.447	1.000
	3	1	−0.112	0.542	1.000
		2	0.054	0.447	1.000

STT-1, Schirmer’s tear Test 1; COS, corneal opacity grade scores.

**Table 4:** Results of (a) ANOVA and (b) Games–Howell post-hoc test for the results of the NIBUT, TFBUT, and STT-1 tests.

(a) Results of ANOVA					
	Sum of squares	Degrees of freedom	Mean square	*F*	*p*-value
Between Tests	3837.494	2	1918.747	41.596	0.000
Within Tests	7334.407	159	46.128		
Total	11171.9	161			

(b) Results of the Games–Howell post-hoc test					
Dependent variable	(I) Tests	(J) Tests	Mean difference (I-J)	Standard error	*p*-value
Results of tests	NIBUT	TFBUT	−3.167^*^	1.441	0.076
		STT-1	−11.537^*^	1.236	0.000
	TFBUT	NIBUT	3.167^*^	1.441	0.076
		STT-1	−8.370^*^	1.234	0.000
	STT-1	NIBUT	11.537^*^	1.236	0.000
		TFBUT	8.370^*^	1.234	0.000

^*^The mean difference is significant at the 0.1 level. NIBUT, non-invasive tear film break-up time, TFBUT, tear film break-up time; STT-1, Schirmer’s tear Test 1.

## 4 Discussion

The precorneal tear film (PTF) is a part of the ocular surface, which includes the conjunctiva, cornea, eyelids, and lacrimal glands. The defense mechanism of the PTF protects the ocular structures, and abnormalities in this function eventually lead to the development of ocular surface disease (OSD). OSD is a concept used to describe a group of diseases, such as blepharitis, meibomian gland dysfunction, allergic eye diseases, and most frequently, and almost synonymously, DED. The dry eye workshop II (DEWS II) conducted by the Tear Film & Ocular Surface Society (TFOS) in 2017 states that the ability to maintain an intact tear film plays a vital role in protecting the ocular surface (1). Several diagnostic methods have been introduced for the evaluation of tear film stability. These methods include the administration of questionnaires, namely the ocular surface disease index (OSDI) and dry eye questionnaire −5 item (DEQ-5), and the evaluation of NIBUT, TFBUT, lipid quality test, and tear meniscus height. An important criterion for diagnosing DED is the results of the questionnaires. However, it is not possible to consider every aspect of DED in veterinary patients as they cannot explain the extent of the clinical symptoms themselves. Several studies in human medicine have defined the severity of DED based on imaging-based tests, in addition to subjective results such as questionnaires (2). These tests have not yet been tailored to veterinary patients; therefore, many reports suggest the application of these tests to veterinary medicine and aim to set the baseline data for animals. Thus, the numerical values to assess NIBUT results of veterinary patients were adopted from reports in human medicine and designated as Group 1, 2, and 3.

In the present study, STT-1, NIBUT, and TFBUT tests were conducted together to evaluate which result held more significance in predicting changes in COS over time. While STT-1 primarily serves as a diagnostic method for evaluating quantitative tear deficiency, both TFBUT and NIBUT assess qualitative tear deficiency. The results of this study confirmed that NIBUT is more reliable in predicting the changes in COS than STT-1 and TFBUT. Some studies on the correlation between TFBUT and NIBUT have been published in human medicine (20, 21). However, the results of these studies varied. NIBUT was longer in one study, whereas TFBUT was longer in another study (20, 21). However, to the best of the authors’ knowledge, no studies have determined which of these two values is more sensitive to the changes in the cornea. It is difficult to find studies comparing NIBUT and TFBUT in veterinary medicine, and relatively similar values were obtained when NIBUT and TFBUT were compared in these studies (11, 22). However, as the values were compared in normal dogs, it is inappropriate to apply them directly in clinical practice.

The examiner must directly observe the drying of the fluorescein dye in the patient’s eye to examine the TFBUT; thus, subjective intervention is inevitable. Moreover, the fluorescein dye used in TFBUT strongly destabilizes the tear film, leading to variability in the volume and density of applied fluorescein, especially with fluorescein-impregnated strips. These factors raise questions about the reproducibility of results in clinical practice, and the stability of tears may be further compromised following a TFBUT examination with fluorescein dye. Given these limitations, we developed this diagnostic method utilizing NIBUT instead of TFBUT. An alternative objective method is NIBUT. One method of examining NIBUT in veterinary ophthalmology is via the use of ICP OSA-VET (OSA; SBM Sistemi, Italy) (4). This device helps analyze DED by evaluating the aqueous, mucin, and lipid layers of tears. This device is relatively easy to use and is associated with good to secure objectivity (14). However, it is relatively expensive and is operated independently from a slit-lamp biomicroscope. Therefore, although examination using a slit-lamp microscope is routinely performed in clinical practice, performing a separate analysis for DED using OSA is considered inconvenient. The grid device of the slit-lamp biomicroscope was used to illuminate the cornea in this study, thereby forming a grid line on the cornea. Videos were obtained subsequently using the digital imaging equipment of the slit-lamp biomicroscope, and analyzed to measure the NIBUT. This method of evaluation using video images can increase the accuracy of the examination. Moreover, it enables the simultaneous evaluation of the entire cornea. With the utilization of artificial intelligence, this device can also enhance the objectification of NIBUT (14).

This study has certain limitations. First, a grid LED light was used in this study. Grid-patterned lines are associated with the disadvantage of the width between the lines in the central and peripheral areas of the cornea appearing different than circular lines. It may be necessary to create a concentric line for more precise inspection. Second, in the significance testing, the confidence interval was verified at a 10% level of significance. It was deemed appropriate to verify the confidence interval within a 10% range in the significance testing, as obtaining a sufficient number of cases that meet the criteria was challenging, leading to a broader confidence interval for the verification. However, it will be necessary to collect more data and verify whether there is significance in the significance test within a 5% level in the future. Third, the follow-up period was not consistent. The duration of the two COS examinations must be the same for all patients to enable accurate evaluation.

Despite various exclusion factors and experimental limitations in this test, the method employed in this study was able to easily obtain 20 s images from general ophthalmic patients. It was validated that this method could serve as a foundation for developing a predictive model for corneal changes. Through this study, we believe that we will be able to diagnose early OSD and establish a predictive model for corneal diseases based on this examination.

In conclusion, using the grid-line illumination plate NIBUT test, eyes with a break-up time of <5 s have a significantly higher chance of having an increased corneal opacity score compared with that of those with a break-up time of >5 s. Among NIBUT, STT-1, and TFBUT, NIBUT was the only test that showed a significant association with the changes in COS. NIBUT can be considered a useful test for determining the status of the ocular surface based on the results of this study. Diligent eye care is recommended for eyes with a NIBUT of <5 s. Further investigations with a larger population in controlled environment are warranted.

## Data availability statement

The original contributions presented in the study are included in the article/Supplementary material, further inquiries can be directed to the corresponding author.

## Ethics statement

The animal studies were approved by Institutional Animal Care and Use Committee of Konkuk University (protocol no.: KU20123). The studies were conducted in accordance with the local legislation and institutional requirements. Written informed consent was obtained from the owners for the participation of their animals in this study.

## Author contributions

SJL: Conceptualization, Formal analysis, Writing – original draft, Writing – review & editing. MGH: Data curation, Formal analysis, Writing – original draft. SJY: Data curation, Formal analysis, Writing – original draft. YSC: Data curation, Formal analysis, Writing – original draft. JYK: Conceptualization, Formal analysis, Project administration, Supervision, Writing – original draft, Writing – review & editing.
